# HYKYHT: A versatile, affordable, and open-source 3D-printed liquid aspiration system

**DOI:** 10.1016/j.ohx.2024.e00509

**Published:** 2024-01-24

**Authors:** Teng-Cheong Ha, Michael Morgan, Axel Schambach

**Affiliations:** a Institute of Experimental Hematology, Hannover Medical School, Hannover, Germany; b REBIRTH Research Center for Translational Regenerative Medicine, Hannover Medical School, Hannover, Germany; c Hannover Biomedical Research School, Hannover, Germany; d Division of Hematology/Oncology, Boston Children's Hospital, Harvard Medical School, Boston, MA, USA

**Keywords:** Suction, Single and multichannel, Tip ejector, Chemical and biological liquid waste, Wet lab

## Abstract

This paper aims to provide the details for making affordable single and multichannel liquid aspirators for wet labs. A liquid aspirator is a basic laboratory device that can cost several hundred Euros. We present a < €25 3D print solution that performs equally well in daily lab routines and is compatible with various vacuum sources, including an aquarium pump or household vacuum cleaner. Presently, commercial aspirators cost more than a decent entry-level 3D printer capable of producing all the parts listed in this manuscript. The models were designed with Tinkercad, with easy printing and minimal support in mind. The versatility and the ultra-low-cost solution we presented could ease the daily workflow of researchers in various research fields. Furthermore, it is valuable to high school or undergraduate student labs and community wet labs for science enthusiasts, where funding is generally limited.

## Graphical abstract

**Figure:**
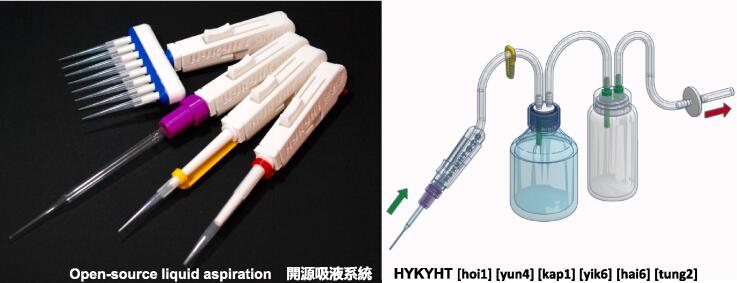

## Specifications table

**Table:** 

Hardware name	[hoi1] [yun4] [kap1] [yik6] [hai6] [tung2] (HYKYHT; Yale Romanization of Cantonese 開源吸液系統) Open-source liquid aspiration system.
Subject area	• Chemistry and biochemistry • Medical (e.g., pharmaceutical science) • Neuroscience • Biological sciences (e.g., microbiology and biochemistry) • Environmental, planetary and agricultural sciences • Educational tools and open source alternatives to existing infrastructure • General
Hardware type	• Biological sample handling and preparation • General liquid waste handling
Closest commercial analog	Corning® Vacuum Aspirator, €461.10 https://web.archive.org/web/20230927140106/https://ecatalog.corning.com/life-sciences/b2b/DE/en/Equipment/Liquid-Handling-Equipment/Corning%C2%AE-Vacuum-Aspirator/p/4930 Integra Biosciences™ Vacuboy Hand Operator, €313.00 https://web.archive.org/web/20230927140617/https://www.fishersci.de/shop/products/p/10565942 VACUUBRAND VHCpro VacuuHandControl, €154.00 https://web.archive.org/web/20230927142306/https://shop.vacuubrand.com/en/vacuuhandcontrol-vhcpro-20688061.html
Open source license	Creative Commons Attribution-Share Alike 4.0 (CC-BY-SA 4.0)
Cost of hardware	< €25
Source file repository	https://doi.org/10.17632/frk98hnwxk.3

## Hardware in context

Removing liquid waste is a simple, but essential daily task in a wet laboratory setting. Each washing step within a given protocol requires the removal of supernatant. Typically, there are three methods for supernatant removal: a) direct pouring from the vessel, b) utilizing pipettes (e.g., Pasteur pipette, micropipette, or pipette aid), or c) employing a vacuum-driven aspirator. The first two methods often fail to achieve complete supernatant removal, potentially leading to interference with subsequent reaction steps.

Using an aspirator can maximize supernatant removal efficiency, but introduces the risk of 1) accidentally discarding the actual samples and 2) potential sample cross-contamination if the same nozzle is reused. Commercial aspiration units commonly feature finger-operated mechanisms to adjust vacuum strength and nozzles compatible with most micropipette tips to mitigate these challenges.

However, commercial aspirators can be prohibitively expensive, costing over €200 (Table 1), and the process of replacing micropipette tips often involves manual removal or suboptimal ejector mechanisms, compromising biosafety and raising contamination risks. Moreover, the option of simultaneous multi-well suction devices is frequently sold separately or bundled for a higher price, increasing expenses for laboratories with diverse research needs. Underfunded laboratories might opt to forgo aspirators, resorting to manual pipetting of liquid waste, which results in an increased workload for researchers and a heightened risk of developing pipetting-related repetitive strain injuries, including conditions such as carpal tunnel syndrome and De Quervain's syndrome [1], [2], [3], [4].

**Table 1:** Commercial handheld aspirator and accessories cost.

Commercial Product	Cost and included accessories	Extra accessories and costs
Corning® Vacuum Aspirator [10]	€461.10 Controller handpiece Pasteur pipette holder Single-channel adapter for disposable pipet tips with ejector	8-channel adapter with tips ejector, €258.40
Integra Biosciences™ Vacuboy Hand Operator [11]	€313.00 VACUBOY hand operator 1-channel stainless adapter 40 mm 1-channel adapter with ejector for disposable tips (with locking) Rubber adapter for Pasteur pipettes Silicone grease	8-channel adapter with tips ejector, €148.00 Stand for VACUBOY Hand operator, €95.50
VACUUBRAND VHCpro VacuuHandControl [12]	€154.00 VHCpro suction controller Single-channel tips adapter without ejector	Adapter for single-channel pipette tips with tips ejector, €46.90 8-channel tip adapter with tips ejector, €358.00 Table stand for VHCpro, €89.80

With the success of the RepRap project [5] in popularizing 3D printers among the general public, the open science community has partially addressed this price gap. In 2016, Marco Ciro published an 8-channel vacuum aspirator (Thing: 1486294) on Thingiverse [6]. Later, Julia Gala de Pablo adapted and modified this into a 6-channel aspirator suitable for 96- and 24-well plates, sharing the model on Printables.com (Model: 399373) [7]. However, these early adaptations include an ejector mechanism that resembles the suboptimal commercial solution.

In this study, we present the concept of a fully 3D-printable open-source liquid aspiration system, aptly named 'HYKYHT' (an abbreviation of '開源吸液系統' in Yale Romanization Cantonese; [hoi1] [yun4] [kap1] [yik6] [hai6] [tung2]). This system seeks to provide an affordable, adaptable, and convenient alternative to commercial aspirators, that caters to laboratories with varying resource levels.

## Hardware description

**Fig. 1:**
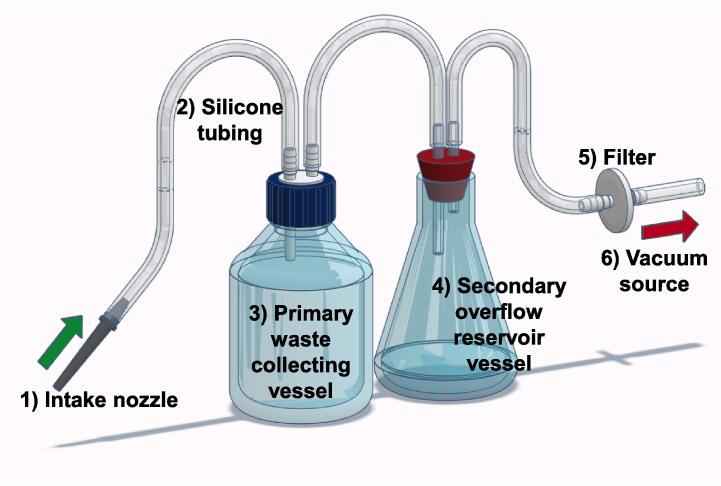
A typical liquid waste aspirator configuration.

A standard liquid waste aspirator configuration comprised the following components: 1) a nozzle, 2) tubing of variable lengths, 3) a primary waste collection vessel, 4) a secondary overflow reservoir vessel, 5) a filter, and 6) a vacuum source (see Fig. 1). Commercial laboratory aspirators often come with a substantial cost, even before inclusion of a vacuum pump and collection vessels. In contrast, the 3D printer employed in prototyping and refining all designs presented in this manuscript can be acquired for less than €150 at the time of manuscript preparation [8].

**Fig. 2:**
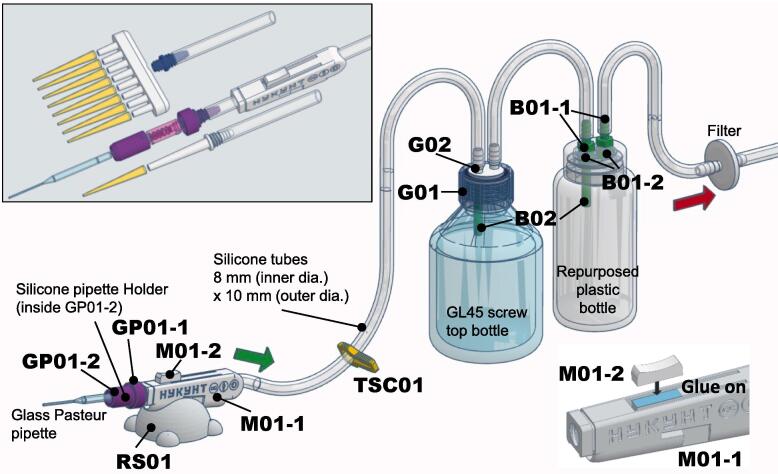
The HYKYHT aspiration system is depicted as a reference for implementation. All parts are labeled in bold using the code numbers listed in Table 2, Table 3 for easy identification. While the primary illustration employs the glass pipette assembly as an example, the boxed illustration offers an overview of all nozzle assemblies, showcasing how the silicone tube passes through the M01-1 unit to establish connections with the nozzles. The bottom right portion of the figure highlights the only obligatory adhesive procedure during assembly, wherein the M01-2 button is attached to the pressure regulator of the M01-1 main handheld unit.

Our 3D-printed solution encompasses components for creating a handheld, pressure-controllable aspirator unit with a nozzle-swapping feature. This unit can be configured as a single-channel, 8-channel, or glass Pasteur pipette adapter (Fig. 2). The single and 8-channel nozzles are compatible with most micropipette tips and incorporate a user-friendly ejection mechanism inspired by micropipettes. Additionally, we have designed 3D-printable GL-45 cap and barb connectors, allowing for the conversion of GL-45 threaded bottles or repurposed buffer bottles from commercial kits into waste collection and reservoir vessels. Our design can be adapted for use with a variety of vacuum sources, offering an easily deployable, versatile, and cost-effective alternative to conventional liquid waste aspirator setups.

Furthermore, the models are generated using Tinkercad (Autodesk, Inc., Mill Valley, California, USA), a freely available web-based computer-aided design (CAD) software that employs a simplified constructive solid geometry approach to construct 3D models [9]. The creation process within Tinkercad is entirely transparent, as ungrouping the models reveals each component and action leading to the final design. Moreover, the shared models are fully customizable, allowing users to modify or improve them.

Our HYKYHT 3D printable liquid waste aspirator design features:

• A low-cost, customizable solution for daily wet lab tasks.
• Tip change convenience, micropipette-inspired tip ejection for easy exchanges and prevention of cross-contamination.
• Tool swap ease with screw-in mechanism for swift exchange between single-channel, 8-channel adapters, and Pasteur pipette holder.
• Versatile mounting for tabletop and wall-mounted aspirator handheld holders.
• Versatile bottle adaptability, GL45 cap with dual barb adapters and individual adapters for converting standard laboratory bottles to waste containers.
• 3D printed mold for casting silicone glass Pasteur pipette holders.

## Design files summary

The links to the stereolithography (STL) files and the design source (Tinkercad) are listed in Table 2 with the description, and the render images of each component are listed in Table 3.

**Table 2:** Name of each design file and sources.

Component file name	File type	Open source license	Location of the file
M01-1_Main handheld unit.stl	STL and source link	CC-BY-SA 4.0	STL: https://doi.org/10.17632/frk98hnwxk.3 Tinkercad: https://s.gwdg.de/w8nQJb
M01-2_Regulator button.stl	STL and source link	CC-BY-SA 4.0	STL: https://doi.org/10.17632/frk98hnwxk.3 Tinkercad: https://s.gwdg.de/kk1u7J
SC01_Single-channel nozzle.stl	STL and source link	CC-BY-SA 4.0	STL: https://doi.org/10.17632/frk98hnwxk.3 Tinkercad: https://s.gwdg.de/2lJDwp
SC02-1_Single-channel nozzle 1000 µL tip ejector arm.stl	STL and source link	CC-BY-SA 4.0	STL: https://doi.org/10.17632/frk98hnwxk.3 Tinkercad: https://s.gwdg.de/lEPSNE
SC02-2_Single-channel nozzle 1000 µL tip ejector.stl	STL and source link	CC-BY-SA 4.0	STL: https://doi.org/10.17632/frk98hnwxk.3 Tinkercad: https://s.gwdg.de/OLmSqp
SC03-1_Single-channel nozzle 200 µL tip ejector arm.stl	STL and source link	CC-BY-SA 4.0	STL: https://doi.org/10.17632/frk98hnwxk.3 Tinkercad: https://s.gwdg.de/KORfsb
SC03-2_Single-channel nozzle 200 µL tip ejector.stl	STL and source link	CC-BY-SA 4.0	STL: https://doi.org/10.17632/frk98hnwxk.3 Tinkercad: https://s.gwdg.de/YPuPWK
MC01-1_8-channel nozzles.stl	STL and source link	CC-BY-SA 4.0	STL: https://doi.org/10.17632/frk98hnwxk.3 Tinkercad: https://s.gwdg.de/g7kpwN
MC01-2_M01-1 8-channel nozzles and MC01-1 handheld unit connector.stl	STL and source link	CC-BY-SA 4.0	STL: https://doi.org/10.17632/frk98hnwxk.3 Tinkercad: https://s.gwdg.de/sjrpqH
MC02-1_8-channel nozzle tips ejector arm.stl	STL and source link	CC-BY-SA 4.0	STL: https://doi.org/10.17632/frk98hnwxk.3 Tinkercad: https://s.gwdg.de/D3HdVJ
MC02-2_8-channel nozzle tips ejector.stl	STL and source link	CC-BY-SA 4.0	STL: https://doi.org/10.17632/frk98hnwxk.3 Tinkercad: https://s.gwdg.de/Odt9cJ
GP01-1_Glass Pasteur pipette's silicone holder housing cap and connector.stl	STL and source link	CC-BY-SA 4.0	STL: https://doi.org/10.17632/frk98hnwxk.3 Tinkercad: https://s.gwdg.de/syVPgd
GP01-2_Glass Pasteur pipette's silicone holder housing.stl	STL and source link	CC-BY-SA 4.0	STL: https://doi.org/10.17632/frk98hnwxk.3 Tinkercad: https://s.gwdg.de/XXpio4
GP02-1_Glass Pasteur pipette silicone holder mold part #1.stl	STL and source link	CC-BY-SA 4.0	STL: https://doi.org/10.17632/frk98hnwxk.3 Tinkercad: https://s.gwdg.de/61MSPT
GP02-2_Glass Pasteur pipette silicone holder mold part #2.stl	STL and source link	CC-BY-SA 4.0	STL: https://doi.org/10.17632/frk98hnwxk.3 Tinkercad: https://s.gwdg.de/hxtDEO
GP02-3_Glass Pasteur pipette silicone holder mold part #3.stl	STL and source link	CC-BY-SA 4.0	STL: https://doi.org/10.17632/frk98hnwxk.3 Tinkercad: https://s.gwdg.de/qmyZLe
GP02-4_Glass Pasteur pipette silicone holder mold part #4.stl	STL and source link	CC-BY-SA 4.0	STL: https://doi.org/10.17632/frk98hnwxk.3 Tinkercad: https://s.gwdg.de/fhJJ7N
RS01_Tabletop stand.stl	STL and source link	CC-BY-SA 4.0	STL: https://doi.org/10.17632/frk98hnwxk.3 Tinkercad: https://s.gwdg.de/jtP3RG
RS02_Wall mount holder.stl	STL and source link	CC-BY-SA 4.0	STL: https://doi.org/10.17632/frk98hnwxk.3 Tinkercad: https://s.gwdg.de/Rl20uT
TSC01_Tubing slide clamp.stl	STL and source link	CC-BY-SA 4.0	STL: https://doi.org/10.17632/frk98hnwxk.3 Tinkercad: https://s.gwdg.de/eX1ZuA
B01-1_Barb connector with male thread.stl	STL and source link	CC-BY-SA 4.0	STL: https://doi.org/10.17632/frk98hnwxk.3 Tinkercad: https://s.gwdg.de/a8rUhv
B01-2_Barb connector hex nut.stl	STL and source link	CC-BY-SA 4.0	STL: https://doi.org/10.17632/frk98hnwxk.3 Tinkercad: https://s.gwdg.de/1O9qri
B02_Extension tube.stl	STL and source link	CC-BY-SA 4.0	STL: https://doi.org/10.17632/frk98hnwxk.3 Tinkercad: https://s.gwdg.de/GoM7Yg
B03_Two-headed barb connector.stl	STL and source link	CC-BY-SA 4.0	STL: https://doi.org/10.17632/frk98hnwxk.3 Tinkercad: https://s.gwdg.de/wQFaAQ
G01_GL45 cap with hole.stl	STL and source link	CC-BY-SA 4.0	STL: https://doi.org/10.17632/frk98hnwxk.3 Tinkercad: https://s.gwdg.de/Ou0aS0
G02_GL45 cap insert with two barb connectors.stl	STL and source link	CC-BY-SA 4.0	STL: https://doi.org/10.17632/frk98hnwxk.3 Tinkercad: https://s.gwdg.de/T4Y2SH
G03_O-ring for GL45 cap insert.stl (TPU; optional)	STL and source link	CC-BY-SA 4.0	STL: https://doi.org/10.17632/frk98hnwxk.3 Tinkercad: https://s.gwdg.de/ljxOdu

**Table 3:** Description for each component and rendered images.

Component rendered image	Description
M01-1: Main handheld unit	The main handheld controller unit of the HYKYHT aspirator enables operators to precisely control suction strength, interchange nozzles effortlessly using a screw-on mechanism, and incorporate an ejection mechanism for micropipette tip removal. Importantly, this component remains isolated from liquid waste contact, serving solely as a conduit for silicone tubing to facilitate pressure regulation.
M01-2: Regulator button	The regulator button comfortably accommodates the operator's thumb or index finger during suction strength adjustment. It is securely attached to the M01-1 main handheld unit using adhesive bonding.
SC01: Single channel nozzle	The single-channel nozzle is compatible with most 200 µL and 1000 µL micropipette tips, directly connecting to the silicone tube. It attaches securely to the M01-1 main unit and comes into direct contact with liquid waste.
SC02-1: Single-channel nozzle 1000 µL tip ejector arm	The single-channel nozzle 1000 µL tip ejector arm is connected to the SC02-2 component by a snap-fit design to create a cohesive ejection unit for removal of 1000 µL micropipette tips from the SC01 nozzle.
SC02-2: Single-channel nozzle 1000 µL tip ejector	See SC02-1
SC03-1: Single-channel nozzle 200 µL tip ejector arm	The single-channel nozzle 200 µL tip ejector arm connected to the SC03-2 component by snap-fit to create a cohesive ejecting unit for removal of 200 µL micropipette tips from the SC01 nozzle.
SC03-2: Single-channel nozzle 200 µL tip ejector	See SC03-1
MC01-1: 8-channel nozzles	The 8-channel nozzle unit is equipped with nozzles spaced to accommodate a standard 96-well plate, enabling the simultaneous removal of liquid waste. These nozzles are compatible with most 200 µL micropipette tips. The unit is designed for use with the MC01-2 connector for attachment to the M01-1.
MC01-2: M01-1 8-channel nozzles and MC01-1 handheld unit connector	This connector joins both the MC01-1 8-channel unit and the M01-1 main unit. Additionally, it also connects to the silicone vacuum tube.
MC02-1: 8-channel nozzle tip ejector arm	The 8-channel nozzle tip ejector arm is connected to the MC02-2 by snap-fit to form an ejecting unit designed to simultaneously eject eight 200 µL micropipette tips from the MC01-1 nozzles.
MC02-2: 8-channel nozzle tip ejector	See MC02-1
GP01-1: Glass Pasteur pipette silicone holder housing cap and connector	This part functions as a connector that links the silicone tube with the silicone holder for the glass Pasteur pipette (either cast using the GP02 mold assembly or obtained commercially). It is securely attached to the M01-1 main unit and paired with the GP01-2 component to house and secure the silicone pipette holder.
GP01-2: Glass Pasteur pipette silicone holder housing	See GP01-1
GP02-1: Glass Pasteur pipette silicone holder mold part #1	GP02-1 to GP02-4 collectively formed a mold for production of a silicone holder to hold a glass Pasteur pipette.
GP02-2: Glass Pasteur pipette silicone holder mold part #2	See GP02-1
GP02-3: Glass Pasteur pipette silicone holder mold part #3	See GP02-1
GP02-4: Glass Pasteur pipette silicone holder mold part #4	See GP02-1
RS01: Tabletop stand	This unit is a tabletop stand that offers a stable platform to hold the aspirator during bench work. It incorporates four holes underneath the stand, specifically designed to insert small neodymium magnets (3 mm in diameter and 2 mm in height). These magnets are securely attached with adhesive to enable the firm attachment of the stand to ferromagnetic surfaces.
RS02: Wall mount holder	This unit is a wall-mounted holder for the aspirator that provides a convenient storage solution. It is equipped with four holes underneath the stand, designed for the insertion and adhesive attachment of small neodymium magnets (3 mm in diameter and 2 mm in height). These magnets allow secure attachment to ferromagnetic walls. Alternatively, the unit can be mounted using glue or double-sided tape.
TSC01: Tubing slide clamp	This component is a clamp for the vacuum-connected silicone tube to effectively halt the airflow and maintain the vacuum. This feature proves particularly valuable when multiple units are connected to a single vacuum pump.
B01-1: Barb connector with male thread	This component is valuable when users choose to create a DIY waste bottle by repurposing plastic buffer bottles commonly found in laboratories. In such cases, two holes with a diameter of 13 mm-14 mm need to be drilled into the bottle cap to accommodate the barb connector, which is secured using the B01-2 hex nut from the inside of the cap. To achieve an airtight seal, hot-melt adhesive or Parafilm can be applied at the interface of the connector and the cap.
B01-2: Barb connector hex nut	See B01-1
B02: Extension tube	The extension tube is an integral part of the barb assembly on the inlet side. Its primary function is to maintain a height differential between the waste inlet and the vacuum outlet, and thus prevent liquid ingress into the dry zone of the apparatus. This component can be easily attached by screwing it onto either B01-1 or G02.
B03: Two-headed barb connector	The two-headed barb connector is designed to extend two shorter silicone tubes.
G01: GL45 cap with hole	The GL45 cap, in combination with the G02 insert and B02 extension tube, converts commonly found GL45 threaded glass bottles into efficient waste collection and overflow reservoir vessels.
G02: GL45 cap insert with two barb connectors	See G01
G03: O-ring for GL45 cap insert (TPU; optional)	The O-ring is an optional component that can be printed using soft, flexible filaments such as TPU. Its purpose is to establish an airtight seal between G01 and G02. However, it is worth noting that securely screwing the G01 onto the bottle automatically presses G02 toward the cap, ensuring adequate sealing of the apparatus.

## Bill of materials (BOM)

Most of the parts for a complete HYKYHT assembly are 3D printable. The non-3D printable components and their prices are listed in Table 4 for reference. Tooling such as a 3D printer, hot glue gun, and driller are not listed as part of BOM. The cost of a vacuum unit is also omitted, as the availability can vary depending on local market and regulatory guidelines.

### Bill of materials summary

**Table 4:** Materials and costs for building a complete HYKYHT aspiration system.

No.	Parts to produce	Parts to obtain	Amount	Unit cost	Consumed cost	Source
1	All 3D printed parts except G03	PLA filaments	142 g for all parts sliced according to Table 5	€13.49 / kg	€1.92	Sunlu.com[13]
2	Silicone tubes for connecting the apparatus	8 mm (inner dia.) x 10 mm (outer dia.) silicone tubes	200 cm, (may vary depending on setup)	€2.56 / meter	€5.12	eBay [14]
3	Waste collection and reservoir bottle	GL45 screw top bottle	2 x 500 mL	€8.00 / bottle	€16.00	LaborXing [15]
4	Repurposed waste collection and reservoir bottle	Empty buffer bottles	2 x 500 mL	€0.00 / bottle	€0.00	Laboratory recycle bin
5	Silicone holder for glass Pasteur pipette	2 components silicon rubber mix	∼ 1.6 mL (∼1.6 g)	€31.90 / kg	€0.05	Amazon [16]
6	Silicone holder for glass Pasteur pipette	Vaseline (Petroleum jelly)	0.5 mL	€3.95 / 50 mL	€0.04	Amazon [17]
7	M01-1 and M01-2	Quick dry super glue	1 drop	€1 / g	€0.05	Local one Euro shop
8	G03-TPU O-ring. (optional)	TPU filaments	1 g	€20.99 / kg	€0.02	Amazon [18]
9	RS01 and RS02(optional)	3 x 2 mm Neodymium magnets	8 pcs (4 pcs for each)	€9.63 / 50 pcs	€1.54	Amazon [19]
				Total:	€8.72 - €24.74	

## Build instructions

### 3D printed parts

The 3D models were generated with Tinkercad, stereolithography (STL) files were exported, sliced using Cura (Ultimaker; Utrecht, Netherlands), and printed with an Ender 2 Pro (Creality 3D; Shenzhen, China) during prototyping. Upon finalization of the models, the final STL files were sliced with Bambu Studio (Bambu Lab GmbH, Frankfurt am Main, Germany) and printed on a X1C or P1P 3D printer (Bambu Lab) for verification.

All models were sliced and printed with 0.4 mm nozzle, 0.2 mm layer height, 3 wall counts, 3 bottom and top layers. While support may be recommended for certain models, it is not required. In cases where a brim is necessary, an additional 0.2 mm height brim layer is included in the STL file; versions without the extra brim layer are also available in the repository for user convenience. Detailed parameters for printing each design can be found in Table 5.

**Table 5:** 3D print parameters for each component.

Design file name	Support	% Infill (Gyroid)	Brim
M01-1_Main handheld unit.stl	No	20 %	No
M01-2_Regulator button.stl	No	20 %	No
SC01_Single-channel nozzle.stl	Rec.	100 %	Yes
SC02-1_Single-channel nozzle 1000 µL tip ejector arm.stl	No	50 %	No
SC02-2_Single-channel nozzle 1000 µL tip ejector.stl	No	100 %	No
SC03-1_Single-channel nozzle 200 µL tip ejector arm.stl	No	50 %	No
SC03-2_Single-channel nozzle 200 µL tip ejector.stl	No	100 %	No
MC01-1_8-channel nozzle.stl	No	50 %	Yes
MC01-2_M01-1 8-channel nozzle and MC01-1 handheld unit connector.stl	Rec.	100 %	Yes
MC02-1_8-channel nozzle tip ejector arm.stl	No	50 %	No
MC02-2_8-channel nozzle tip ejector.stl	No	50 %	No
GP01-1_Glass Pasteur pipette silicone holder housing cap and connector.stl	Rec.	100 %	Yes
GP01-2_Glass Pasteur pipette silicone holder housing.stl	No	100 %	No
GP02-1_Glass Pasteur pipette silicone holder mold part #1.stl	No	20 %	No
GP02-2_Glass Pasteur pipette silicone holder mold part #2.stl	No	20 %	No
GP02-3_Glass Pasteur pipette silicone holder mold part #3.stl	No	20 %	No
GP02-4_Glass Pasteur pipette silicone holder mold part #4.stl	No	20 %	No
RS01_Tabletop stand.stl	No	20 %	No
RS02_Wall mount holder.stl	No	20 %	No
TSC01_Tubing slide clamp.stl	No	20 %	No
B01-1_Barb connector with male thread.stl	Rec.	100 %	Yes
B01-2_Barb connector hex nut.stl	No	100 %	No
B02_Extension tube.stl	No	100 %	Yes
B03_Two-headed barb connector.stl	No	100 %	Yes
G01_GL45 cap with hole.stl	No	50 %	No
G02_GL45 cap insert with two barb connectors.stl	No	50 %	No
G03_O-ring for GL45 cap insert.stl (TPU; optional)	No	100 %	No

Rec.: recommended. Note to readers: print orientation and parameters of all models were sliced and printed without issues using Cura and Bambu Studio. However, some slicers (or different versions of the used slicers) might prompt potential adhesion problems on certain models, which can be ignored after careful inspection.

The total print time using the above recommendations (without support) to print each of the listed components (except G03) with PLA is estimated to be 6 h and 2 min by Bambu Studio (Ver. 1.8.2.56) on their X1C and P1P printers. In contrast, Cura (Ver. 5.4) estimates 20 h and 13 min for the Ender 2 standard profile, distributed across two build plates due to the bed size limitation. Please note that these numbers are for reference only, as different slicers, printers, print materials, and actual print times may vary.

Caution should be taken during 3D printing, as it involves high temperatures and mechanical movement. In addition, 3D printed parts may contain sharp edges and can break when excessive force is applied, particularly during removal from the print bed.

### Overview of HYKYHT aspiration system assembly.

Users can customize the HYKYHT aspiration system to suit their specific laboratory requirements. For instance, users should consider the most prevalent laboratory applications, including the types of culture ware typically used. Based on these considerations, the appropriate nozzle(s) and ejection mechanism can be selected for 3D printing and assembly.

Furthermore, users must decide between employing a GL45 screw-top glass bottle or repurposing a plastic bottle as the primary collecting vessel and the secondary overflow reservoir vessel to choose which barb assembly to print. Fig. 2 provides a reference for the implementation of the aspiration system.

Lastly, the selection of a vacuum source is crucial. Laboratories equipped with professional vacuum pumps likely have established methods for connecting the pump to a silicone tube. Alternatively, users may opt for an alternative vacuum source, such as a household vacuum cleaner or aquarium pump. However, it is imperative that users thoroughly review and adhere to their local laboratory safety guidelines when making this decision.

For those contemplating the use of an alternate vacuum source, Ziv Botzer from Israel provides a valuable resource—a customizable tool for generating 3D printable adapters for household vacuum cleaners, accessible on Ultimaker's Thingiverse (thing:1571860) [20].

### HYKYHT handheld assembly

**Fig. 3:**
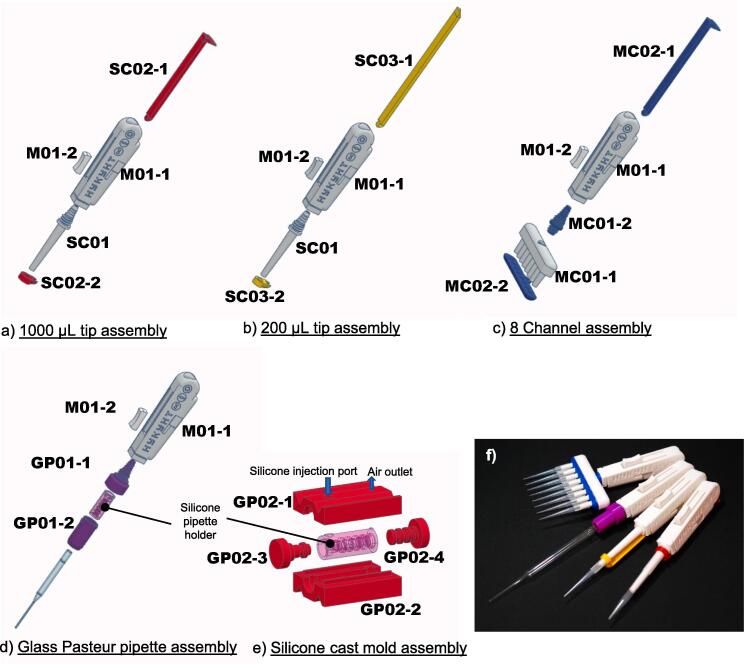
Expanded view of the individual handheld assembly options (a-d), e) silicone pipette holder mold assembly, and f) photo of the completed handheld units.

The HYKYHT handheld unit consists of the versatile M01-1 main unit, which accommodates the passage of a silicone tube (Fig. 2, upper left) for vacuum strength control. For secure assembly, a finger rest button (M01-2) is designed to be attached to the M01-1, as illustrated in the lower-right section of Fig. 2. Various handheld assemblies are shown in detail in Fig. 3 a-d.

**Fig. 4:**
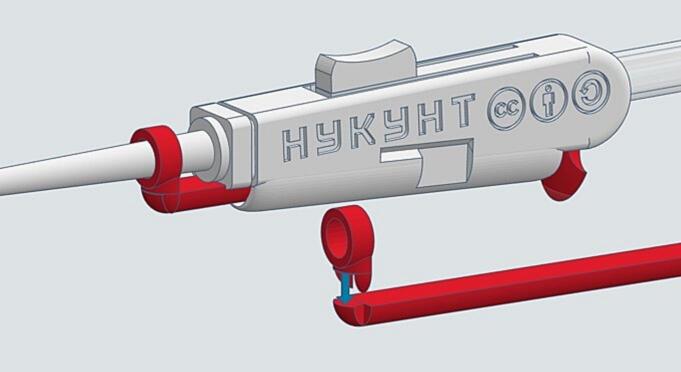
A close-up view of the snap joint of the 1000 µL ejector assembly. Similar snap joints are also applied to the 200 µL and 8-channel ejector assemblies.

To assemble single-channel micropipette tips, the single nozzle, SC01, is connected to the silicone tube (Fig. 2, upper left) before being screwed onto the M01-1 main unit (Fig. 3 a, b). Depending on the desired configuration, a 1000 µL or 200 µL tip ejector mechanism can then be attached (Fig. 3 a, b, f) by snap-fitting the ejector arm with the ejector (Fig. 4). In the case of assembling the 1000 µL ejector, SC02-1 and SC02-2 must be assembled before screwing the SC01 onto the M01-1 unit.

To create the 8-channel assembly, the connector MC01-2 is first threaded onto the MC01-1 nozzles, followed by the secure attachment of the silicone tube to the other end of MC01-2. The assembly is then fastened onto the M01-1 handheld unit. The ejector components are subsequently installed by snap-fitting the MC02-1 and MC02-2 (Fig. 3c).

Alternatively, users have the option to choose a glass Pasteur pipette assembly. This assembly requires the use of a silicone pipette holder (commercially available or produced following the instructions in the following section) to fit onto an internal nozzle within GP01-1, which is then enclosed within GP01-2. The vacuum silicone tube is connected to the other end of GP01-1 before being secured onto the handheld assembly (Fig. 3d).

### Casting the silicone glass pipette holder

To fabricate the silicone pipette holder for the glass Pasteur pipette assembly, we employed silicone mold-making rubber, commonly utilized by mold-making enthusiasts. This material typically consists of two liquid components, namely part A and part B, which must be blended according to the manufacturer's specified ratio before being poured into a mold for casting. Subsequently, the mixture is left to undergo a curing process, during which it transforms from a liquid silicone blend into a solid, rubber-like object.

#### Application of petroleum jelly as a mold release agent

Prior to casting the silicone rubber, a thin layer of a mold release agent is applied to the mold to ensure the subsequent release of the casted object without causing damage. To achieve this, a thin coating of petroleum jelly is evenly spread onto the inner surfaces of GP02-1 and GP02-2, as well as the projecting structures of GP02-3 and GP02-4, which form the internal cavity of the silicone pipette holder. Excess coating should be removed as thoroughly as possible. A small brush can be employed to apply and distribute the petroleum jelly evenly.

#### Assembly of the casting form

**Fig. 5:**
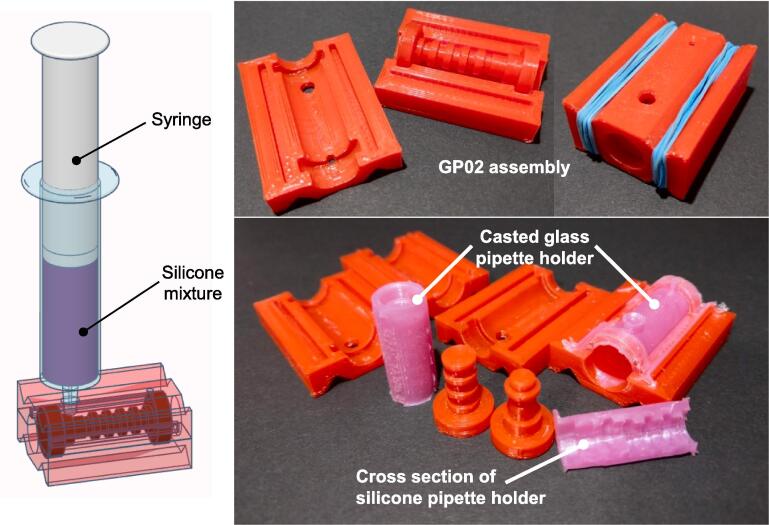
Tinkercad rendered image depicting the injection of silicone mixture into the GP02 assembly and photography of the GP02 assembly with the silicone casted Pasteur pipette holder.

The assembly of the casting form is illustrated in Fig. 3e. In summary, the assembly process involves inserting GP02-3 and GP02-4 into the designated grooves of GP02-2, followed by securely snapping GP02-1 into place to create the final assembly. Notably, the assembly features two V-shaped grooves on both sides, which are for rubber bands or binder clips to hold the mold firmly in position (Fig. 5). This ensures the structural integrity of the mold during the subsequent step of injecting the silicone blend.

#### Preparation of the silicone rubber mix

Mix components (A and B) according to the manufacturer's instructions. Given that the cavity volume of the GP02 mold is approximately 1600 mm^3^, a final blend volume of 2 mL of silicone rubber is adequate for a single cast. During the mixing process, air bubbles tend to form within the silicone mixture. These entrapped air bubbles can compromise the structural integrity of the final product, diminishing the sealing effectiveness of the pipette holder. To address this issue, a syringe-based method is employed for air bubble removal:

1) The silicone mixture is drawn into a syringe of suitable size (3 mL syringe in this case).
2) While maintaining the syringe upright, a vacuum is applied by retracting the syringe plunger and blocking the syringe nozzle.
3) The applied pressure is then released slowly, causing air to accumulate at the top of the syringe near the nozzle opening.
4) Repeat steps 2 and 3 until no visible air bubbles remain in the silicone blend.
5) Unblock the nozzle and carefully remove any accumulated air at the top of the syringe.

This process effectively minimizes the presence of air bubbles within the silicone blend, ensuring the production of a high-quality pipette holder with optimal sealing properties.

#### Injection of silicone mixture into GP02 casting mold

Upon preparing the silicone blend, proceed with injecting it into the GP02 casting mold using the following steps:

1) Begin by injecting half of the silicone blend slowly into the GP02 casting mold through the larger inlet opening (as illustrated in Fig. 3e and 5). Silicone blends typically possess a certain viscosity; therefore, to prevent air pockets within the mold, it is crucial to allow the blend to flow gradually and continuously.
2) Gently tap the mold a few times on a hard surface. This tapping action helps ensure the even distribution of the silicone mixture and aids in the removal of any trapped air pockets within the mold.
3) Proceed to inject the second half of the silicone blend. It is normal for some of the blends to overflow from the smaller outlet opening. Continue to tap the mold gently after this injection step.

Check for any signs of leakage from the sides of the mold. If leakage occurs, carefully examine the integrity of the mold. Parafilm tape can seal potential leakage points, but should not cover the openings of the mold.

Then, follow the manufacturer's instructions for curing the casted mold. Typically, a curing period of 24 h is sufficient. The silicone blend solidifies and takes on the desired shape during this time. After the curing period, carefully disassemble the mold to release the silicone casted glass pipette holder. Exercise caution during this step to avoid damaging the final product.

### Assembling liquid waste and overflow reservoir bottles

There are two main ways to make waste collection bottles compatible with the HYKYHT aspirator. If decontaminating the liquid waste is mandatory, we strongly suggest using the GL45 bottle that allows autoclaving. However, caution has to be made when autoclaving the bottle, as the 3D-printed parts cannot be autoclaved. The original cap for the glass bottle has to be used during autoclaving. Alternatively, a cheaper version can be employed by preparing a repurposed plastic buffer container of sufficient strength to avoid collapse and withstand vacuum pressure.

#### GL45 bottles

**Fig. 6:**
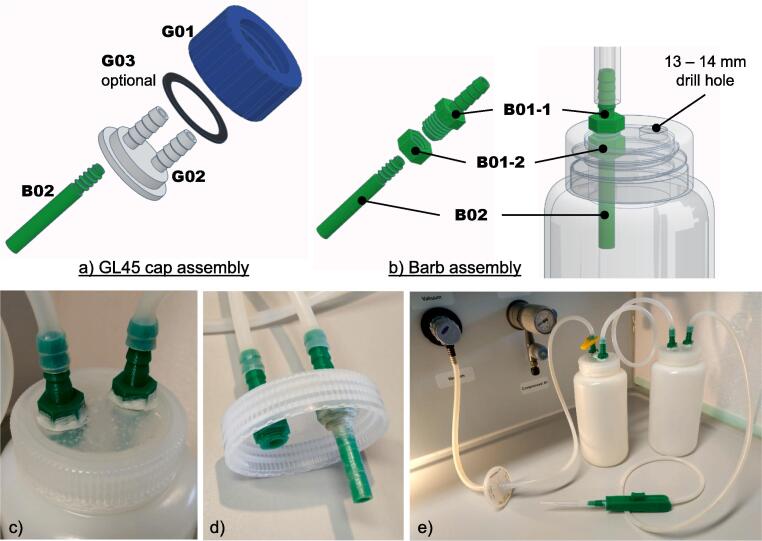
Expanded view of reservoir bottle assembly with (a) GL45 cap assembly for GL45 glass bottle or (b) barb assembly for repurposed plastic bottle. Examples of the barb assembly on the cap of a repurposed plastic bottle (c-d) and (e) a complete assembly of an early HYKYHT prototype are displayed for reference.

For this approach, G01, G02, and B02 must be printed. Optionally, G03 can be printed (TPU) for better sealing. The printed cap assembly are shown in Fig. 6a; insert G02 into G01, G03, if printed, should be placed in between G01 and G02. B02 is then screwed to the bottom of G02 to create a difference between the height of the barb assembly's waste inlet and vacuum direction. After assembly, remove the original GL45 cap from the bottle to replace it with the assembly.

#### Repurposed plastic bottles

For a less expensive setup, plastic bottles from reagent kits can be repurposed. To do this, drill two 13 – 14 mm holes on the cap. Pass B01-1 through the drilled hole and screw it tight with B01-2 to secure the barb assembly on the cap (Fig. 6b), then screw B02 to one of the barb assembly. Parafilm tape or hot-melt-glue can be applied around the barb and cap interface to seal any leaking points (Fig. 6c-d). An early prototype using repurposed plastic bottles is shown in Fig. 6e.

### Assembling tabletop and wall-mounted holder

**Fig. 7:**
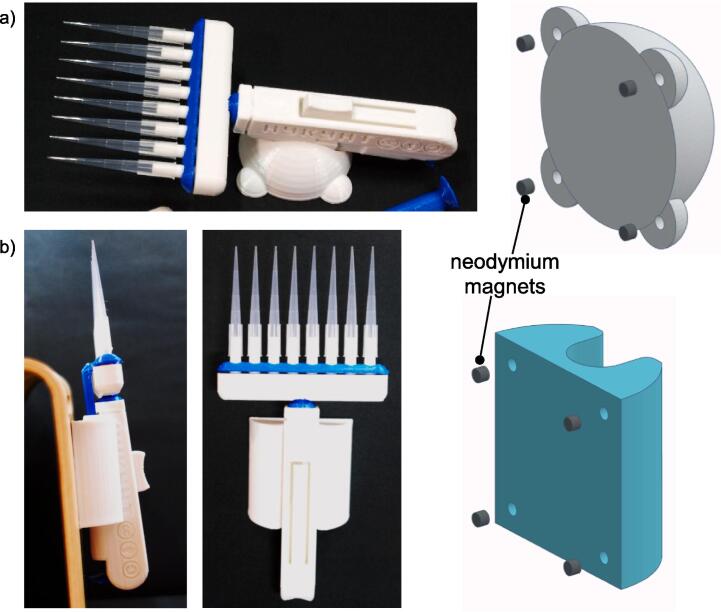
Example of a multichannel handheld stored on (a) tabletop stand and (b) wall mount holder. Neodymium magnets with 3 mm diameter and 2 mm thickness can be attached to the designated holes underneath these parts.

The tabletop (RS01) and the wall-mounted (RS02) holders are designed to store the handheld when not in use. These parts can be printed and used directly. However, small neodymium magnets can be glued into the four holes (3 × 2 mm) underneath the part for attachment to ferromagnetic surfaces (Fig. 7).

## Operation instructions

Fig. 2 displays the complete assembly of the HYKYHT aspiration system. This system functions similarly to commercial aspiration devices. Unlike most commercial handheld aspirators, the HYKYHT system maintains constant maximum suction. To adjust suction strength, users can press the regulator button on the handheld, decreasing airflow and thus suction power. Releasing the button increases the flow rate. This design choice follows the “keep it simple, stupid” (KISS) design principle to simplify the 3D printing process and reduce the need for intricate components.

A tubing clamp (TSC01) can be positioned anywhere on the silicone tubing to interrupt airflow for an extended duration. All nozzle types can be easily exchanged using a screw-on mechanism, and tip ejectors feature a snap-fit assembly.

For safety and optimal performance, we strongly recommend several features when assembling the aspiration system:

1) Utilize two bottles, one for waste collection and a second for overflow prevention.
2) Incorporate the B02 extension tube to adjust the height of the inlet and outlet openings in both bottles.
3) Include a 0.45 µm (or smaller pore size) disc filter before the tubing connection to the vacuum source. This filter acts as a biological barrier to prevent contaminants or larger particles from reaching and damaging the vacuum source.

Please be aware that using an aspiration device can generate aerosols. It is advisable to wear personal protective equipment (PPE), including safety goggles, gloves, and a laboratory coat, when using the apparatus.

While it is technically possible to use affordable vacuum sources like an aquarium pump or a household vacuum cleaner, compliance with local regulations is essential when deploying the HYKYHT system.

During the prototyping and testing phase, all 3D-printed parts were produced with PLA (except for the optional G03 O-ring). However, operators can select different build materials to suit their specific needs, especially considering the chemical compatibility of the reagents used in their laboratory. For reference, Heikkinen et al. have conducted tests and compiled information on the chemical compatibility of commonly used 3D printing polymers and laboratory reagents [21].

## Validation and characterization

The PLA-printed HYKYHT prototype was used in our laboratory for over 18 months, subjecting them to real-world conditions without significant wear and tear. These parts have consistently withstood the application of alcohol-based solvents for nozzle decontamination with no signs of degradation.

The HYKYHT system features screw-on connections for most components, but the tip ejector parts utilize a snap joint design. These tip ejectors are especially vulnerable because they are routinely subjected to pressure when ejecting tips. To assess their durability, we conducted tests by repeatedly applying and ejecting micropipette tips with the HYKYHT aspirator unit. This process continued until the tip ejection mechanism failed or until we exhausted 10 boxes of micropipette tips (960 times for 200 µL and 1000 µL tip ejectors and 120 times for 8-channel ejectors). Three ejecting units of each type (SC02, SC03 and MC02) were printed and tested. Notably, none of the ejectors failed the test, highlighting the effectiveness and durability of the snap joint design and 3D-printed parts.

## Ethics statements

This project does not involve human subjects or animal experiments.

## CRediT authorship contribution statement

**Teng Cheong Ha:** Conceptualization, Data curation, Formal analysis, Funding acquisition, Investigation, Methodology, Project administration, Resources, Validation, Visualization, Writing – original draft, Writing – review & editing. **Michael Morgan:** Supervision, Writing – review & editing. **Axel Schambach:** Funding acquisition, Project administration, Resources, Supervision, Writing – review & editing.

## Declaration of competing interest

The authors declare that they have no known competing financial interests or personal relationships that could have appeared to influence the work reported in this paper.
